# Controllable rheological properties of UV-responsive calix[4]arene gel for drug encapsulation and release

**DOI:** 10.1039/d4ra06787h

**Published:** 2024-12-11

**Authors:** Ji Ha Lee, Wataru Kanda, Tomoyuki Tachibana, Minhye Kim, Sung Ho Jung, Riku Kawasaki, Akihiro Yabuki

**Affiliations:** a Institute for Fiber Engineering (IFES), Faculty of Textile Science and Technology, Shinshu University 3-15-1 Tokita Ueda 386-8567 Japan leejiha@shinshu-u.ac.jp +81 26 821 5571; b Chemical Engineering Program, Graduate School of Advanced Science and Engineering, Hiroshima University 1-4-1 Kagamiyama Higashi-Hiroshima 739-8527 Japan; c Department of Chemistry, Research Institute of Advanced Chemistry, Gyeongsang National University Jinju 52828 Republic of Korea; d Applied Chemistry Program, Graduate School of Advanced Science and Engineering, Hiroshima University Higashi-Hiroshima 739-8527 Japan

## Abstract

This study investigates the potential of calix[4]arene-based supramolecular gels for use in drug delivery systems, focusing on both their rheological properties and controlled drug release behavior. We explore how key factors, including temperature, solvent exchange, and UV exposure, influence the gel's mechanical strength and its ability to encapsulate and release drugs. Specifically, our work examines how these external stimuli affect the stability of the gel matrix and modulate the release rate of the encapsulated drug. By systematically evaluating the effects of each factor, we aim to identify conditions that optimize drug release kinetics. The findings offer valuable insights into the development of a tunable, responsive platform for efficient drug delivery, highlighting the potential of calix[4]arene gels as promising candidates for advanced therapeutic applications.

## Abstract 2

This study investigates the potential of calix[4]arene-based supramolecular gels for use in drug delivery systems, focusing on both their rheological properties and controlled drug release behavior.

## Graphical abstract

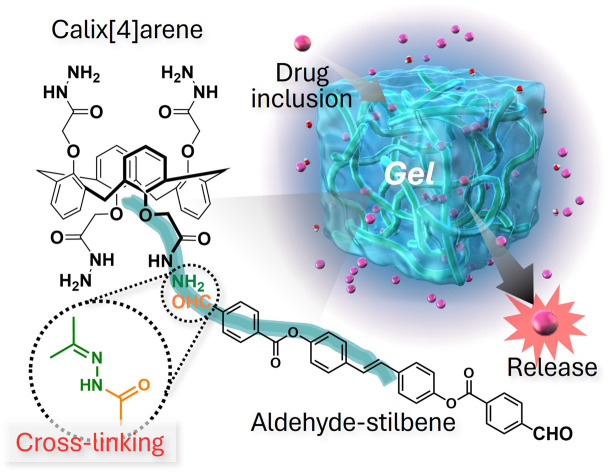

## Introduction

Gel materials, distinguished by their formation of three-dimensional (3D) networks through aggregation, have garnered substantial attention owing to their exceptional mechanical properties and diverse applications spanning fields including food science, agriculture, sensing technology, tissue engineering, catalysis, and pharmaceuticals.^1–4^ Our research mainly focuses on harnessing gels as efficient drug delivery channels within the pharmaceutical sector. The distinctive characteristics of gels enable them to undergo versatile transformations, transitioning from soft materials to elastic and stiff materials, depending on factors such as solvent composition, concentration, and molecular interactions.^5–8^ Leveraging these structural changes, we anticipate diverse applications of gels as carriers in drug delivery systems. Exploration into stimuli-responsive gels responsive to external stimuli plays a pivotal role in tailoring the mechanical properties of gels and unlocking new functionalities. External stimuli, predominantly influenced by environmental conditions such as temperature, pH, and light, serve as the focal points of this research.^9–11^ By investigating innovative methodologies to manipulate the structure and properties of gels, we aim to expand their scope of applications across various fields. Previous studies conducted by our research group explored supramolecular gels, including crown ether, calixarene, and cyclodextrin.^12–14^ Notably, our investigations have led to the development of a crown ether-based metal-coordinated supramolecular gel with distinct properties. Additionally, we have reported on the mechanical strength of a calixarene-based supramolecular gel under various environmental conditions.^15,16^ Recent research has explored the suitability of gel materials as drug carriers, establishing a close relationship between the rheological properties of gels and drug release, and the development of gel materials for drug delivery.^17,18^ These advancements in gel-based drug delivery systems have underscored the pivotal role of rheological properties in modulating drug release behaviour. Understanding how changes in rheology influence drug release mechanisms is crucial for optimizing gel-based formulations for effective drug delivery applications. This study investigates the rheological changes in a calix[4]arene gel in response to various external stimuli, including UV exposure, solvent exchange, and drug inclusion and release ratios of drugs.

## Results and discussion

### Synthesis and morphological analysis of calix[4]arene gels: effects of temperature on gel formation and structure

**Fig. 1:**
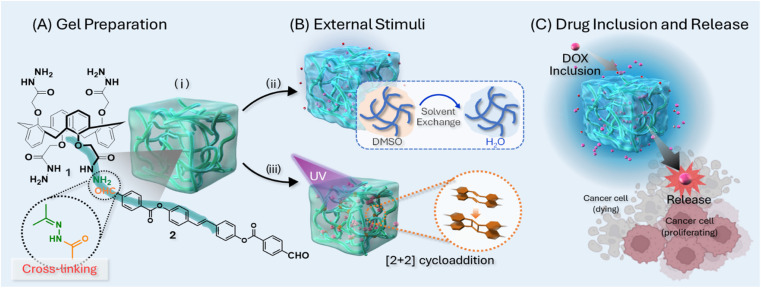
Illustration of this work: (A) gel preparation using calix[4]arene 1 and aldehyde-stilbene 2, (B) the gel subjected to each external stimulus (i) heated at 100 °C, (ii) heated at 100 °C and solvent exchange, (iii) heated at 100 °C and UV exposure (C) drug inclusion using DOX as a model anticancer drug to the prepared gels and its release for action on the cancer cell.

**Fig. 2:**
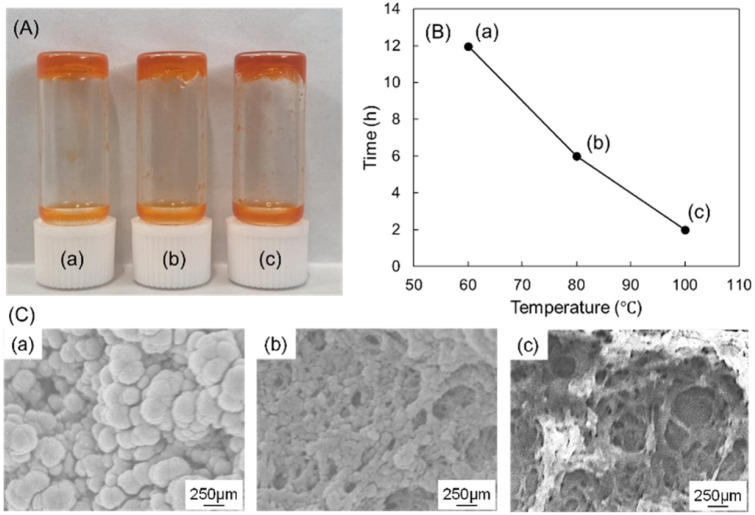
(A) Photograph of DOX-encapsulated gels (B) plot of gelation time of synthesized gels at different temperature (C) SEM images of DOX-encapsulated gels synthesized at (a) 60 °C, (b) 80 °C, and (c) 100 °C.

As illustrated in Fig. 1, hydrazine-based calix[4]arene 1 and a stilbene moiety containing an aldehyde functional group 2 were synthesized following a previously reported procedure.^15,16^ The formation of calix[4]arene gels was achieved by reacting hydrazine and aldehyde in an organic solvent (Fig. 1). The effects of external stimuli such as temperature, solvent exchange, and UV exposure on the calix[4]arene gel were investigated to elucidate the relationship between rheological strength and drug inclusion and release ratio of DOX (doxorubicin hydrochloride), a model anticancer drug. The formation of the calix[4]arene gels was evaluated at different temperatures, and the gelation time decreased under high-temperature conditions. Specifically, at a reaction temperature of 60 °C, gel formation took at least 12 h. However, the gels formed within 6 h at 80 °C and 2 h at 100 °C (Fig. 2). The morphology of the gels prepared at different temperatures was investigated using scanning electron microscopy (SEM Fig. 2C). SEM images of the gel at 60 °C showed a spherical structure with diameters of 2–4 μm. When the gel was obtained at 80 °C, it was characterized by interconnected particles. SEM images of the gel obtained at 100 °C revealed fiber-like structures. This substantial change in morphology is likely due to the increasing hydrazone moiety, leading to a tightly connected network structure with increasing temperature.

### Rheological properties of calix[4]arene gels *via* external stimuli

**Fig. 3:**
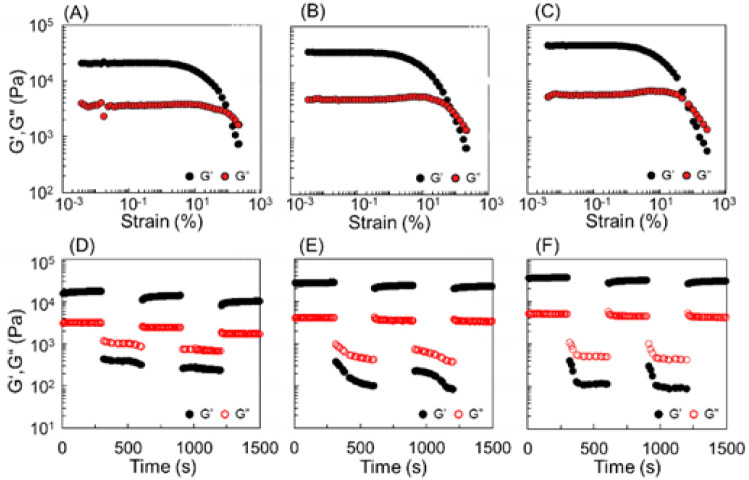
Rheological property of DOX-encapsulated gels synthesized at (A and D) 60 °C, (B and E) 80 °C, and (C and F) 100 °C.

The storage (*G*′) and loss (*G*′′) moduli of the calix[4]arene gels prepared at different temperatures were determined by rheological measurements (Fig. 3A–C: strain; Fig. 3D–F: thixotropy). With the temperature increasing from 60 °C to 80 °C and 100 °C during gelation by the hydrazone reaction, a strain sweep was performed as a function of the amplitude of deformation on shear, with a strain amplitude ranging from 0.001% to 10% at 1 rad s^−1^, respectively. Both the in-phase *G*′ and the out-of-phase *G*′′ remained constant up to approximately 0.1% strain (*G*′ > *G*′′), defining the upper boundary of the linear viscoelastic region (Fig. 3A–C).

**Fig. 4:**
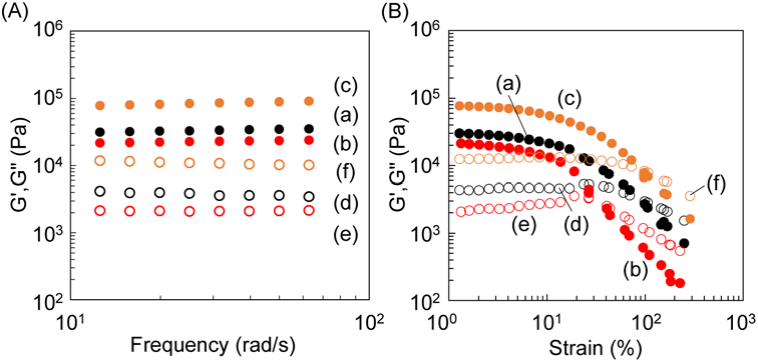
Rheological property of (A) frequency sweep and (B) strain sweeps tests using gels subjected to each external stimulus: (a and d) heat at 100 °C (b and e) heat at 100 °C and solvent exchange (c and f) heat at 100 °C and UV exposure at 256 nm (*G*′: filled circle, *G*′′: unfilled circle).

As the temperature increased, both *G*′ and *G*′′ values exhibited an upward trend. Strain amplitude sweeps of the samples demonstrated an elastic response typical of gels. In the region where *G*′/*G*′′ > 1, the gel predominantly displayed an elastic character. As the strain percentage increased, the transition from *G*′/*G*′′ > 1 to *G*′/*G*′′ < 1 occurred. The reversal points of the strain percentage showed a decreasing tendency with increasing temperature (Fig. 3A–C. 60 °C, reverse point of strain percentage: 107%; 80 °C, reverse point of strain percentage: 82%; 100 °C, reverse point of strain percentage: 76%). We observed the dominance of the elastic response, *G*′, across all frequencies, and each of the supramolecular gels exhibited immediate recovery of *G*′ and *G*′′ in repeated step strain tests alternating above and below their critical strain amplitudes (Fig. 3D–F). To investigate the effect of solvent exchange and UV exposure on the gel, we prepared calix[4]arene gels under various conditions, as shown in Fig. 1. Three types of calix[4]arene gel samples were prepared: (i) gel prepared at 100 °C, (ii) gel prepared at 100 °C and subjected to solvent exchange with water, and (iii) gel prepared at 100 °C and exposed to 256 nm UV light. The *G*′ and *G*′′ values of the gels were investigated using frequency sweeps test mode from 0.628 to 62.8 rad s^−1^, where significant changes in the *G*′ and *G*′′ values were observed, respectively (Fig. 4). Solvent exchange from DMSO to water decreased the *G*′ and *G*′′ values of the gel, whereas, exposure to 256 nm UV light increased the *G*′ and *G*′′ values. The transparent gel became opaque after solvent exchange from DMSO to water because the calix[4]arene gel is hydrophobic.

### UV irradiation effect of calix[4]arene gels

**Fig. 5:**
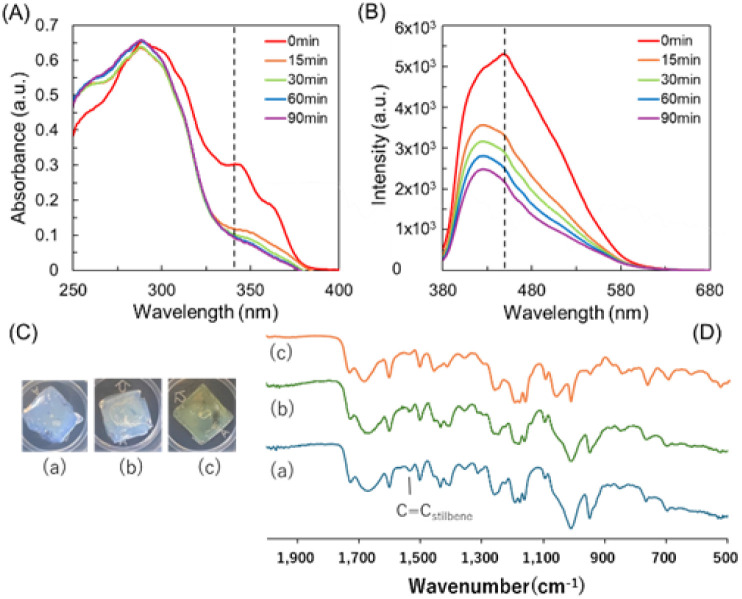
(A) UV spectra of the prepared gel after UV irradiation for different times. (B) Fluorescence spectra of the prepared gel after UV irradiation at 256 nm. (C) Photograph and (D) IR spectra of the prepared gel (a) before and after UV irradiation at (b) 365 nm and (c) 256 nm.

UV exposure at 256 nm causes cyclization of stilbenes,^15^ which lowers the density of the gel and eventually increases the *G*′ and *G*′′ values. The 2 + 2 cyclization reaction was confirmed by UV and fluorescence changes (Fig. 5). Even when exposed to 256 nm for only 15 min, 2 + 2 cyclization occurred, and at this time, the fluorescence around 430 nm rapidly decreased. Each gel was irradiated with 256 nm and 365 nm UV wavelengths, consecutively. Upon irradiation at 256 nm, a [2 + 2] cycloaddition reaction was induced, resulting in a yellow color change on the gel surface. No visible change occurred in the gel irradiated at 365 nm. IR measurements of each gel revealed that the C=C peak of stilbene observed at 1517 cm^−1^ remained unchanged after 365 nm irradiation, whereas the peak disappeared following irradiation at 256 nm.

**Fig. 6:**
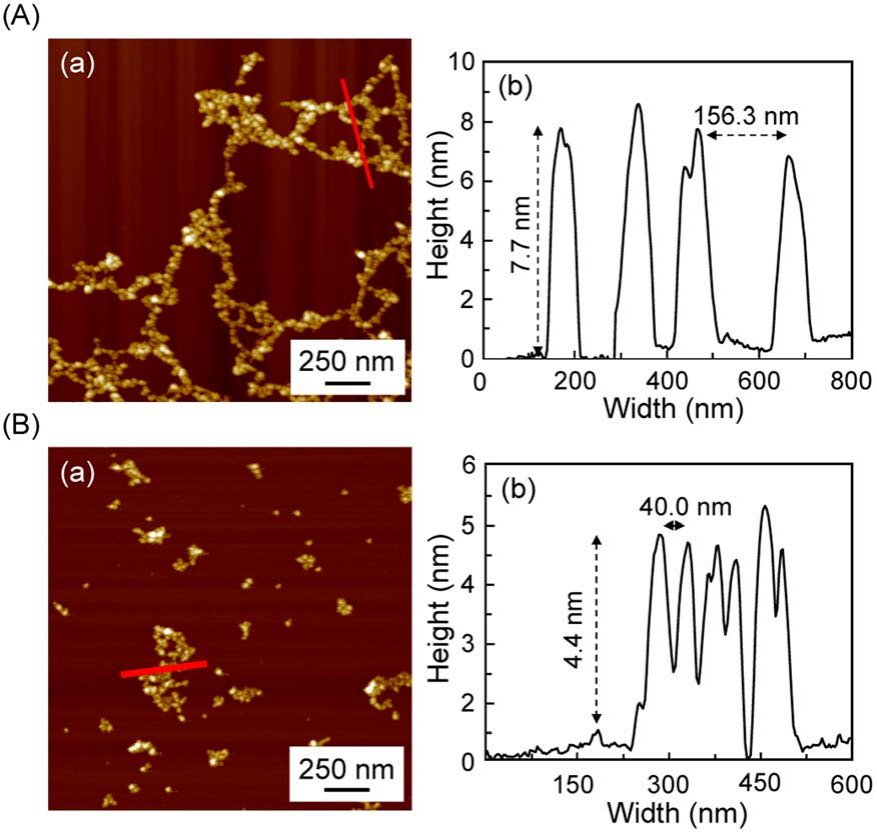
AFM images of gels subjected to each external stimulus: (A) heat at 100 °C (B) heat at 100 °C and UV exposure at 256 nm: (a) AFM images; (b) height of prepared gels shown as a red line in (a).

Although observed at a concentration lower than that used for gel formation, atomic force microscopy (AFM) analysis revealed a notable reduction in the particle size within the network structure from approximately 7–8 nm to 4–5 nm following UV irradiation. Moreover, the spacing between particles decreased, indicating a denser packing of the network structure after irradiation (Fig. 6). This is believed to be induced by a cyclization reaction between stilbenes, as shown in Fig. 5.

### Cytotoxicity test of calix[4]arene gels *via* external stimuli and drug encapsulation and release evaluation

**Fig. 7:**
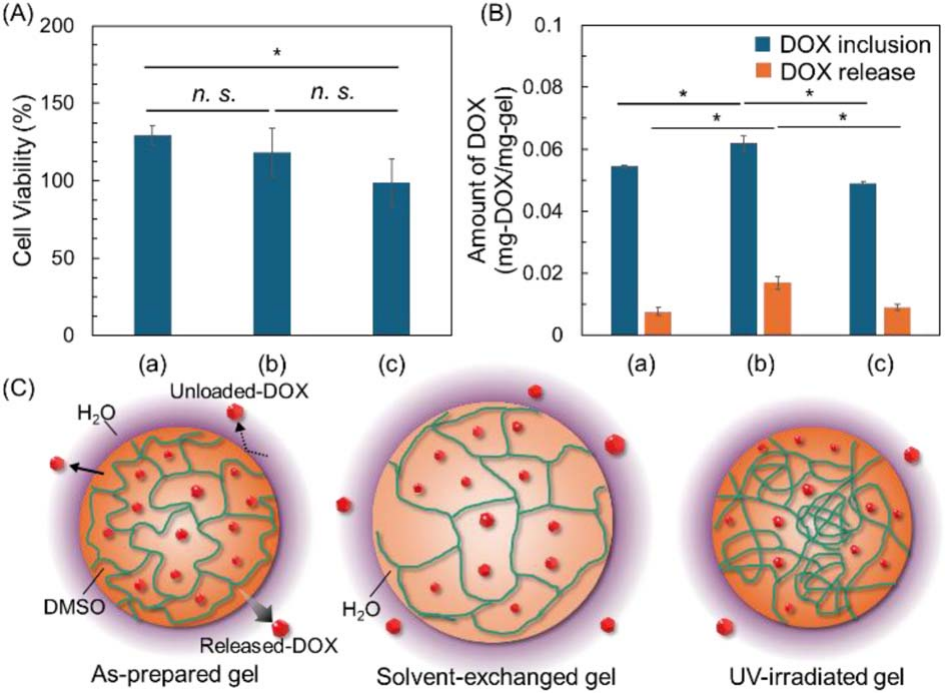
(A) Cytotoxicity of gels toward murine fibroblast L929 cells subjected to each external stimulus: (a) heat at 100 °C (b) heat at 100 °C and solvent exchange (c) heat at 100 °C and UV exposure at 256 nm, statistical significance: *p < 0.05 based on Tukey–Krammer test (*n* = 3). (B) The amount of DOX inclusion and release of gels subjected to each external stimulus: (a) heat at 100 °C (b) heat at 100 °C and solvent exchange (c) heat at 100 °C and UV exposure at 256 nm. (C) Mechanism of DOX inclusion and release of each prepared gels. Statistical significance: *p < 0.05 based on Tukey–Krammer test (*n* = 3). Statistical significance: *p < 0.05 based on Tukey–Krammer test (*n* = 3).

To assess the cytotoxicity of the gel, we conducted a cell viability assay using murine fibroblast cells (L929) with gels prepared under different conditions without DOX (Fig. 7A). The results indicate that gels prepared without DOX showed no apparent cytotoxicity, indicating that the synthesized gel systems are suitable as nontoxic drug release platforms for normal cells under current conditions. Subsequently, the drug inclusion and release amounts of the calix[4]arene gel prepared under each condition (i, ii, and iii) were confirmed (Fig. 7B and Table 1). Interestingly, the percentages of drug inclusion ranged from 72% to 85%; while, the release percentage ranged from 16% to 29%. Although the encapsulation amount exceeded 70%, the released amount of the encapsulated drug was less than 30%. DMSO and water are both relatively small molecules, but water molecules are slightly smaller than DMSO molecules. Therefore, during solvent exchange from DMSO to water in a gel, the smaller water molecules are expected to increase the internal solvent space of the gel (Fig. 7C). The smaller size of water molecules enables them to occupy the same space more efficiently than DMSO molecules. Consequently, solvent exchange to water is generally expected to slightly increase the internal solvent space of the gel, thereby increasing the drug encapsulation and release ratios. Conversely, UV irradiation decreased drug encapsulation and increased drug release. This outcome was anticipated due to the 2 + 2 cycloaddition that induced partial crosslinking inside the gel, resulting in a non-uniform entanglement or loosely crosslinked structure. This structure is believed to impede drug encapsulation, leading to a slight decrease, while drugs encapsulated in the loosely crosslinked regions are easily released, thereby increasing the release ratio.

**Table 1:** DOX inclusion and release ratios in gels subjected to each external stimulus: (i) heat at 100 °C; (ii) heat at 100 °C and solvent exchange; (iii) heat at 100 °C and UV exposure. *n* = 3

	(i)	(ii)	(iii)
DOX inclusion (%)	76.7 ± 6.8	72.3 ± 5.1	85.8 ± 5.8
DOX release (%)	16.3 ± 4.6	17.6 ± 3.6	29.0 ± 3.6

## Experimental

### Materials and methods

Hydrazine-calix[4]arene derivative (compound 1) and aldehyde-bearing stilbene derivative (compound 2) were synthesized according to our previously reported procedures.^1,2^ Dimethyl sulfoxide (DMSO, >99.0% purity) and doxorubicin hydrochloride (DOX, >99.0% purity), the model anticancer drug were purchased from FUJIFILM Wako Pure Chemical Corp. (Japan) and used without further purification. IR spectra were measured in the range of 400 to 4000 cm^−1^ using FT-IR (8400S, Shimadzu Corp.).

### Gel preparation

Compound 1 (2.5 mg, 3.51 μmol) and 2 (3.3 mg, 6.93 μmol) were dissolved in DMSO of 50 and 200 μL in a vial, respectively. These solvents of 1, 2 were heated for 30 min at 100 °C on a bell jar-type oven (BV-001, Shibata Scientific Technology Ltd). Each solvent of 1 and 2 were mixed in a 10 × 10 mm quartz cell and heated at temperatures ranging from 60 to 100 °C for 24 h in a bell jar-type oven to form a gel sample.

### UV irradiation

A wavelength of 256 nm for 3 h was used to irradiate the gel samples by a hand-held UV lamp (Handy UV Lamp, As one Corporation).

### Solvent exchange of gel sample

The prepared gels were immersed in water (3 mL, pH 7) for 1 min at room temperature. The water was replaced after 1 min and the procedure repeated 10 times to change the solvent of the gel samples from DMSO to water.

### Rheological properties of gel

The rheological properties were carried out by using an AR-2000ex (MCR102; Anton Paar GmbH, Graz, Austria). The gel samples were loaded on a rheometer plate according to the standard level. A 20 mm diameter parallel plate was used. The gap between the gels and the plate was 1.0 mm and the experiments were performed at 25 °C. Strain sweep tests were performed by increasing the amplitude of oscillation from 0 to 500% of the apparent shear strain. Frequency sweeps were performed from 0.628 to 62.8 rad s^−1^.

### AFM measurement

Compound 1 (1.25 mg, 1.76 μmol) and Compound 2 (1.67 mg, 3.47 μmol) were dissolved in DMSO of 100 and 400 μL in a vial and the 10 μL removed solvent of diluted with DMSO to 3 mL. The reaction mixture was dropped (10 μL) onto a silicon wafer (1 × 1 cm). After 24 h, the gel formed on the silicon wafer was exposed to UV light for 2 h.

### UV-vis measurement

The gel samples were prepared using a 1 × 10 mm quartz cell in the same way and at different concentrations as explained in gel sample preparation procedure. The gel samples were irradiated with UV light using a hand-held UV lamp for specific time and UV spectra measured in the range of 200 to 700 nm at a rate of 200 nm min^−1^ using a spectrophotometer (V-550, JASCO, Japan).

### PL measurement

The gel samples were prepared using a 20 × 20 mm quartz cell in the same way and concentration as in gel preparation. The gel samples were irradiated with UV light for a specific time and fluorescence analysis was carried out using a fluorescence spectrophotometer (FP8500, JASCO, Japan). Fluorescence analysis was performed with an excitation light of 340 nm at a rate of 200 nm min^−1^ in the range of 380 to 750 nm.

### Biosafety test of prepared gel

Biosafety of the gels were investigated toward murine fibroblast cells (L929, healthy cell). The cells were seeded on 96 well plate at a density of 5.0 × 10^3^ cells per well and incubated overnight (approximately 18 h). The prepared gels were prepared by dropping either 10 μL or 1000 μL of the protein solution without DOX onto 96 well plate and keeping 25 °C for 48 h. After addition of Cell Counting Kit-8, the cell viability was quantified by measuring absorbance at 450 nm using microplate reader.

### Drug inclusion of DOX from gel

DOX (0.4 mg, 0.69 μmol) dissolved in 50 μL of DMSO was added to the gels in the vials. After 24 h, the solvent of the DOX remaining in the vial was removed and diluted to 3 mL with water. Diluted solvent of DOX was measured using a V-550 spectrophotometer (JASCO, Japan) in the range of 200–700 nm at a 200 nm min^−1^ to calculate the amount of unloaded DOX to the prepared gels. From these results, the amount and ratio of loaded DOX in the prepared gels was calculated.

### Drug release of DOX from gel

Water (3 mL, pH 7) was added to the DOX-loaded gels to release the DOX. After 24 h, the DOX released water was gathered from the vial and analyzed by UV-vis. The amount and ratio of DOX released from the DOX-loaded gels were determined using a standard curve of DOX. A standard curve of DOX was produced at a wavelength of 500 nm.

## Conclusions

In conclusion, hydrazine-based calix[4]arene gels demonstrated good responses to temperature, solvent exchange, and UV exposure, revealing significant adaptability in their physical properties and drug delivery capabilities. Temperature elevation shortened gelation times and altered gel morphology from spherical to fiber-like structures, enhancing their mechanical strength. UV exposure particularly increased the gels' stiffness and drug release ratios due to stilbene cyclization, suggesting their applicability in stimuli-responsive drug delivery systems. These findings underscore the potential of gels as versatile, non-toxic platforms for controlled drug release, modifiable through external stimuli. Our comprehensive investigation illuminates the intricate interplay between rheological properties, external stimuli, and drug release behaviour in calix[4]arene gel-based drug delivery systems, offering valuable insights for designing and optimizing such systems in pharmaceutical applications.

## Data availability

The data supporting the findings of this study are available from the corresponding author upon reasonable request. Additional materials, such as raw data, may also be provided upon request, subject to approval.

## Conflicts of interest

There are no conflicts to declare.
